# Nomogram prediction model for renal anaemia in IgA nephropathy patients

**DOI:** 10.1515/med-2021-0284

**Published:** 2021-05-07

**Authors:** Fei Li, Ri-bao Wei, Yang Wang, Ting-yu Su, Ping Li, Meng-jie Huang, Xiang-mei Chen

**Affiliations:** School of Medicine, Nankai University, No. 94 Weijin Road, Nankai District, Tianjin 300073, People’s Republic of China; Department of Nephrology, First Medical Center of Chinese PLA General Hospital, Nephrology Institute of the Chinese People’s Liberation Army, State Key Laboratory of Kidney Diseases, National Clinical Research Center for Kidney Diseases, Beijing Key Laboratory of Kidney Disease Research, No. 28 Fuxing Road, Haidian District, Beijing 100853, People’s Republic of China

**Keywords:** IgA nephropathy, anaemia, chronic kidney disease, nomogram

## Abstract

In this study, we focused on the influencing factors of renal anaemia in patients with IgA nephropathy and constructed a nomogram model. We divided 462 patients with IgA nephropathy diagnosed by renal biopsy into anaemic and non-anaemic groups. Then, the influencing factors of renal anaemia in patients with IgA nephropathy were analysed by least absolute shrinkage and selection operator (LASSO) regression and multivariable logistic regression, and a nomogram model for predicting renal anaemia was established. Eventually, nine variables were obtained, which are easy to apply clinically. The areas under the receiver operating characteristic (ROC) curve and precision-recall (PR) curve reached 0.835 and 0.676, respectively, and the C-index reached 0.848. The calibration plot showed that the model had good discrimination, accuracy, and diagnostic efficacy. In addition, the C-index of the model following internal validation reached 0.823. Decision curve analysis suggested that the model had a certain degree of clinical significance. This new nomogram model of renal anaemia combines the basic information, laboratory findings, and renal biopsy results of patients with IgA nephropathy, providing important guidance for predicting and clinically intervening in renal anaemia.

## 1 Introduction

IgA nephropathy is currently the most common glomerular disease worldwide, with a high incidence in the Asia-Pacific region [1], and is the most common cause of chronic kidney disease (CKD) in China [2]. Renal anaemia is a common diagnostic complication in patients with CKD, in whom it can occur at the early stage (stages 2 and 3 according to the KDIGO guidelines). The level of haemoglobin decreases when the estimated glomerular filtration rate (eGFR) is approximately 70 mL/min/1.73 m^2^ in males and 50 mL/min/1.73 m^2^ in females; however, most commonly, renal anaemia appears in stage 4 CKD and worsens as the disease progresses. Among patients with more advanced disease and those on dialysis, approximately 90% have anaemia [3,4].

The pathogenesis of renal anaemia is complex; the primary mechanism is the reduced production of erythropoietin (EPO) by the paratubular apparatus. In addition, excessive expression of hepcidin, a persistent microinflammatory state, and uraemic toxins have an effect on anaemia [5,6,7,8]. Anaemia because of CKD not only affects the quality of life of the patients but also increases the incidence of and mortality from cardiovascular complications, which may lead to further deterioration of renal function and form a vicious cycle called “cardiac anaemia syndrome” [9]. In addition, renal anaemia has been identified as an independent risk factor of heart failure in CKD patients [10].

Therefore, the early diagnosis of and intervention for renal anaemia is necessary, but the individualized prediction of IgA nephropathy complicated by renal anaemia has been rarely reported and is an urgent problem to be solved. The aim of this study was to establish the first nomogram model for the personalized prediction of the risk of IgA nephropathy complicated by renal anaemia to guide clinical screening of high-risk groups and develop more targeted clinical decisions.

## 2 Methods

### 2.1 Study sample collection and management

A total of 658 patients with IgA nephropathy diagnosed by renal biopsy were enrolled from January 2015 to January 2016. The method of inclusion and exclusion was based on a previous study [11]. Finally, 462 patients were enrolled, including 132 patients with anaemia and 320 patients without anaemia. All patients who underwent renal biopsy signed a study protocol informed consent form for the renal clinical database at the time of admission, agreeing that their data would be used for the clinical study. The study protocol was approved by the hospital ethics committee (EC No.: S2019-309-01).

### 2.2 Definition of clinical measures

According to WHO recommendations, anaemia can be diagnosed in males aged ≥15 years with haemoglobin <130 g/L or in adult non-pregnant females with haemoglobin <120 g/L in regions at sea level [12].

To evaluate renal function, we referred to the National Kidney Foundation Kidney Disease Outcomes Quality Initiative clinical practice guidelines for classifying subjects into different stages of CKD based on eGFR [13], which was calculated using the Chronic Kidney Disease Epidemiology Collaboration (CKD-EPI) equation for Asian populations (CKD stage 1: eGFR ≥ 90 mL/min/1.73 m^2^; CKD stage 2: eGFR: 60–89 mL/min/1.73 m^2^; CKD stage 3: eGFR: 30–59 mL/min/1.73 m^2^; CKD stage 4: eGFR: 15–29 mL/min/1.73 m^2^; and CKD stage 5: eGFR < 15 mL/min/1.73 m^2^) [14]. In this study, CKD stages 1–2, 3, and 4–5 were used as a group, respectively.

The 2017 updated Oxford pathological evaluation criteria [15] were used to identify the type of IgA nephropathy as follows: mesangial hypercellularity (M, M0 ≤ 0.5, M1 > 0.5), endocapillary hypercellularity (E, E0: no, E1: yes), segmental glomerulosclerosis (S, S0: no, S1: yes), tubular atrophy/interstitial fibrosis (T, T0: 0–25%, T1: 26–50%, T2: >50%), and cellular/fibrocellular crescent (C, C0: no, C1: <25% of the glomeruli, C2: ≥25% of the glomeruli).

### 2.3 Statistical analysis

The study design and statistical analysis of this study were carried out in strict accordance with the TRIPOD statement for prediction models [16]. All data analysis was performed using R software (version 3.6.3; https://www.R-project.org). All clinically collected data (basic patient information, laboratory tests, pathological stage for renal biopsy) were enumeration data and are expressed as frequency (%). Least absolute shrinkage and selection operator (LASSO) regression was performed by the “glmnet” package in R software, the “rms” package was used to draw the nomogram and calibration curve, the “pROC” package was used to illustrate the receiver operating characteristic (ROC) curve, the coords function was used to return the values of the variables used in the calculation of the ROC curve, the “modEvA” package was used for plotting the precision-recall (PR) curve, and the “rmda” package was used to draw the decision curve analysis (DCA) curve. LASSO regression is a compression estimation regression method. Its greatest advantage lies in the fact that by performing penalized regression on all variable coefficients, the coefficient of the most relatively insignificant independent variable becomes zero, so that it is excluded from the modelling, improving the modelling stability, and solving the problem of having highly correlated variables in the traditional model [17]. Therefore, LASSO regression was used to screen for possible factors of renal anaemia in IgA patients. Multivariable logistic regression analysis was performed by entering the variables screened by the LASSO regression, and the β regression coefficient, 95% confidence interval, odds ratio (OR), and *P*-value were calculated (statistical significance was assessed bilaterally). A nomogram prediction model was developed based on the results of logistic regression analysis [18], and all potential predictors were used in the development of the model [19]. A calibration curve was drawn to assess the calibration of the renal anaemia prediction model, where a fit closer to the ideal model indicated a better prediction. The predictive accuracy and diagnostic performance of the validated model were quantified using the areas under the ROC and PR curves (AUCs) and Harrell’s concordance index (C-index) [20]. The DCA curve was used to quantify the net benefit at different threshold probabilities in IgA nephropathy cohorts and determine the clinical role of the nomogram in avoiding the possibility of false positives and false negatives [21]. The nomogram model was internally validated by repeating bootstrap resampling 1,000 times [22], and the relative corrected C-index was calculated. For all statistical data, *P* < 0.05 was considered statistically significant. As there were a few missing values for some variables in the data, we assumed that the data were missing at random. In R software, we performed multiple interpolations for the missing values by chained equations [23].

## 3 Results

### 3.1 General information

A total of 462 patients with IgA nephropathy were divided into an anaemia group (132 cases) and a non-anaemia group (330 cases). Table 1 presents all data of the patients in both groups, including demographic information and clinical laboratory tests and pathological examination results.

**Table 1:** Differences between demographic and clinical characteristics of anaemia and non-anaemia groups

Demographic characteristics	*n* (%)		
	Anaemia (*n* = 132)	Non-anaemia (*n* = 330)	Total (*n* = 462)
**Age (years)**			
18–44	92 (69.7)	258 (78.2)	350 (75.8)
45–59	31 (23.5)	67 (20.3)	98 (21.2)
≥60	9 (6.8)	5 (1.5)	14 (3.0)
**Sex**			
Male	58 (43.9)	214 (64.8)	272 (58.9)
Female	74 (56.1)	116 (35.2)	190 (41.1)
**BMI (kg/m** ^**2**^)			
<24	74 (56.1)	135 (40.9)	209 (45.2)
24–27.9	42 (31.8)	131 (39.7)	173 (37.4)
≥28	16 (12.1)	64 (19.4)	80 (17.3)
**Systolic (mm Hg)**			
90–109	16 (12.1)	24 (7.3)	40 (8.7)
110–119	17 (12.9)	54 (16.4)	71 (15.4)
120–129	34 (25.8)	81 (24.5)	115 (24.9)
130–139	18 (13.6)	67 (20.3)	85 (18.4)
140–149	19 (14.4)	57 (17.3)	76 (16.5)
≥150	28 (21.2)	47 (14.2)	75 (16.2)
**Diastolic (mm Hg)**			
<70	14 (10.6)	14 (4.2)	28 (6.1)
70–89	33 (25.0)	70 (21.2)	103 (22.3)
80–89	35 (26.5)	124 (37.6)	159 (34.4)
90–99	27 (20.5)	68 (20.6)	95 (20.6)
≥100	23 (17.4)	54 (16.4)	77 (16.7)
**Serum albumin (g/L)**			
≤30	8 (6.1)	2 (0.6)	10 (2.2)
>30	124 (93.9)	328 (99.4)	452 (97.8)
**24 h protein excretion (g/day)**			
<1	37 (28.0)	151 (45.8)	188 (40.7)
≥1	95 (72.0)	179 (54.2)	274 (59.3)
**Cholesterol (mmol/L)**			
<5.72	118 (89.4)	295 (89.4)	413 (89.4)
≥5.72	14 (10.6)	35 (10.6)	49 (10.6)
**Triglyceride (mmol/L)**			
<1.7	87 (65.9)	170 (51.5)	257 (55.6)
≥1.7	45 (34.1)	160 (48.5)	205 (44.4)
**Serum uric acid (μmol/L)**			
<420	83 (62.9)	216 (65.5)	299 (64.7)
≥420	49 (37.1)	114 (34.5)	163 (35.3)
**BUN (mmol/L)**			
≤7.5	100 (75.8)	258 (78.2)	358 (77.5)
>7.5	32 (24.2)	72 (21.8)	104 (22.5)
**CKD stages**			
1–2	57 (43.2)	263 (79.7)	320 (69.3)
3	48 (36.4)	64 (19.4)	112 (24.2)
4–5	27 (20.5)	3 (0.9)	30 (6.5)
**M** a			
M0	57 (43.2)	203 (61.5)	260 (56.3)
M1	75 (56.8)	127 (38.5)	202 (43.7)
**E** b			
E0	117 (88.6)	277 (83.9)	394 (85.3)
E1	15 (11.4)	53 (16.1)	68 (14.7)
**S** c			
S0	32 (24.2)	110 (33.3)	142 (30.7)
S1	100 (75.8)	220 (66.7)	320 (69.3)
**T** d			
T0	35 (26.5)	185 (56.1)	220 (47.6)
T1	28 (21.2)	96 (29.1)	124 (26.8)
T2	69 (52.3)	49 (14.8)	118 (25.5)
**C** e			
C0	72 (54.5)	197 (59.7)	269 (58.2)
C1	50 (37.9)	125 (37.9)	175 (37.9)
C2	10 (7.6)	8 (2.4)	18 (3.9)

a M, mesangial hypercellularity;

b E, endocapillary hypercellularity;

c S, segmental glomerulosclerosis.

d T, tubular atrophy/interstitial fibrosis;

e C, cellular/fibrocellular crescent.

### 3.2 Screening for factors associated with renal anaemia in patients with IgA nephropathy

**Figure 1:**
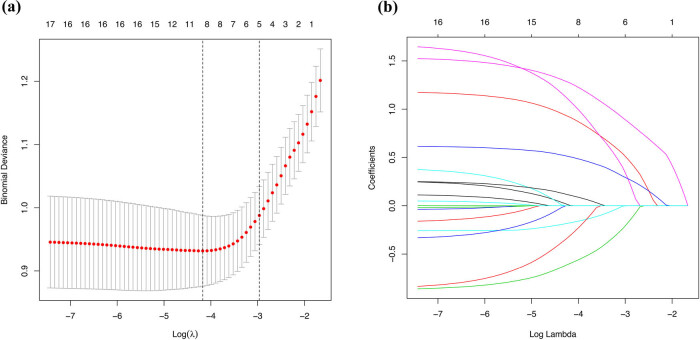
Demographic information, clinical laboratory tests, and pathological examination selection using the LASSO binary logistic regression model. (a) Optimal parameter (lambda) selection in the LASSO model used 10-fold cross-validation via minimum criteria. The partial likelihood deviance (binomial deviance) curve was plotted versus log (lambda). Dotted vertical lines were drawn at the optimal values using the minimum criteria and the 1 SE of the minimum criteria (the 1-SE criterium). (b) LASSO coefficient profiles of the 17 features. A coefficient profile plot was produced against the log (lambda) sequence. Vertical line was drawn at the value selected using 10-fold cross-validation, where the optimal lambda resulted in nine features with non-zero coefficients. Abbreviations: LASSO, least absolute shrinkage and selection operator; SE, standard error.

A total of 17 potential risk factors for renal anaemia were included in this study. The 17 variables were reduced by the LASSO regression reducing dimension algorithm, and representative risk factors for renal anaemia were selected. The lambda parameter value with the smallest 10-fold cross-validation error was used as the optimal value for the model, and the number of variables with a non-zero regression coefficient was also counted (Figure 1a and b). The results of the LASSO regression revealed the following nine variables as risk factors affecting the development of renal anaemia in patients with IgA nephropathy: age, sex, diastolic blood pressure (DBP), serum albumin (ALB), cholesterol (CHOL), triglyceride (TG), CKD stage, mesangial hypercellularity (M), and tubular atrophy/interstitial fibrosis (T).

### 3.3 Logistic regression analysis

The results of logistic regression analysis using age, sex, DBP, ALB, CHOL, TG, CKD stage, M, and T are shown in Table 2.

**Table 2:** Prediction factors for anaemia in IgA nephropathy

Intercept and variable	Prediction model		
	*β*	Odds ratio (95% CI)	*P*-value
Intercept	−1.5434	0.214 (0.072–0.604)	0.004
**Age (years)**			
18–44			
45–59	0.0006	1.001 (0.516–1.894)	0.998
≥60	1.6285	5.096 (1.293–21.958)	0.022
**Sex**			
Male			
Female	1.143	3.136 (1.822–5.516)	<0.001
**Diastolic (mm Hg)**			
<70			
70–89	−0.2236	0.780 (0.291–2.262)	0.667
80–89	−1.0336	0.356 (0.131–0.988)	0.043
90–99	−0.8154	0.442 (0.152–1.304)	0.135
≥100	−1.3312	0.264 (0.078–0.881)	0.03
**Serum albumin (g/L)**			
>30			
≤30	1.6019	1.602 (0.967–37.905)	0.074
**Cholesterol (mmol/L)**			
<5.72			
≥5.72	−0.8782	0.416 (0.156–1.013)	0.064
**Triglyceride (mmol/L)**			
<1.7			
≥1.7	−0.8493	0.428 (0.241–0.742)	0.003
**CKD stages**			
1–2			
3	1.1118	3.040 (1.511–6.176)	0.002
4–5	3.7456	42.333 (10.943–220.631)	<0.001
**M** a			
M0			
M1	0.2739	1.315 (0.770–2.237)	0.313
**T** b			
T0			
T1	0.182	1.200 (0.595–2.380)	0.605
T2	1.425	4.158 (1.838–9.546)	0.001

a M, mesangial hypercellularity;

b T, tubular atrophy/interstitial fibrosis.

### 3.4 Construction of the nomogram prediction model and judgement of diagnostic efficacy

**Figure 2:**
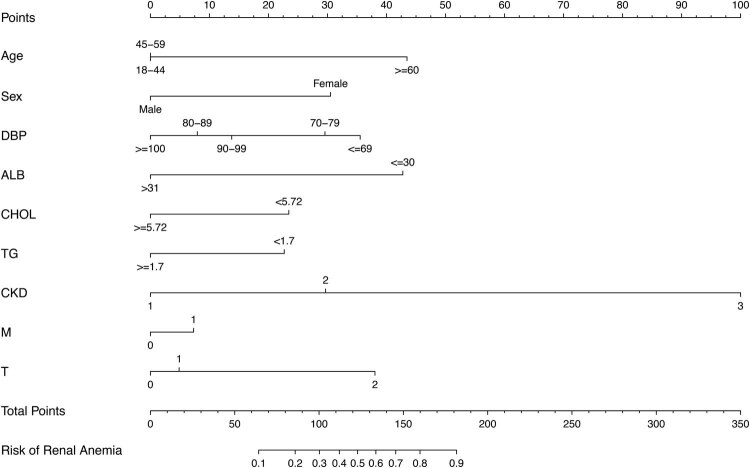
Developed IgA nephropathy patients complicated with renal anaemia nomogram. The renal anaemia nomogram was developed in the cohort, with nine variables, age, sex, DBP, ALB, CHOL, TG, CKD stage, M, and T. Abbreviations: CKD 1, stages 1–2 CKD; CKD 2, stage 3 CKD; CKD 3, stages 4–5 CKD; DBP, diastolic blood pressure; ALB, serum albumin; CHOL, cholesterol; TG, triglyceride; CKD stage, chronic kidney disease stage; M, mesangial hypercellularity; T, tubular atrophy/interstitial fibrosis.

**Figure 3:**
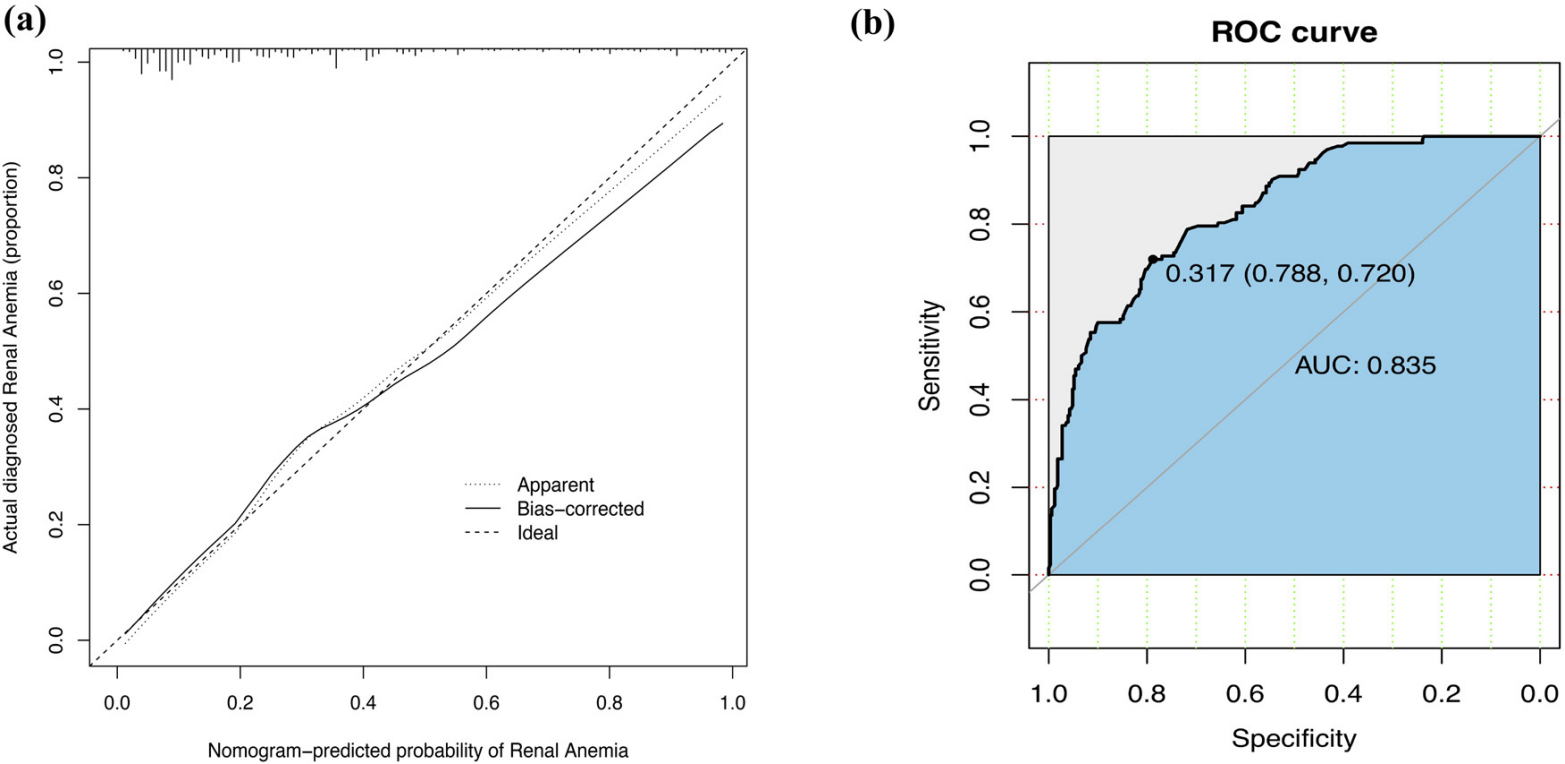
Calibration curve and ROC curve for the renal anaemia nomogram prediction in the cohort. (a) Calibration curve. The *x*-axis represents the predicted renal anaemia risk. The *y*-axis represents the actual diagnosed renal anaemia. The diagonal dotted line represents a perfect prediction by an ideal model. The solid line represents the performance of the nomogram, of which a closer fit to the diagonal dotted line represents a better prediction. (b) ROC curve. It showed that the AUC of this model for predicting renal anaemia was 0.835. The optimal cut-off value of the ROC curve was 0.317, corresponding to a specificity and sensitivity of 0.788 and 0.720, respectively. Abbreviations: ROC curve, receiver operating characteristic curve; AUC, area under the curve.

**Figure 4:**
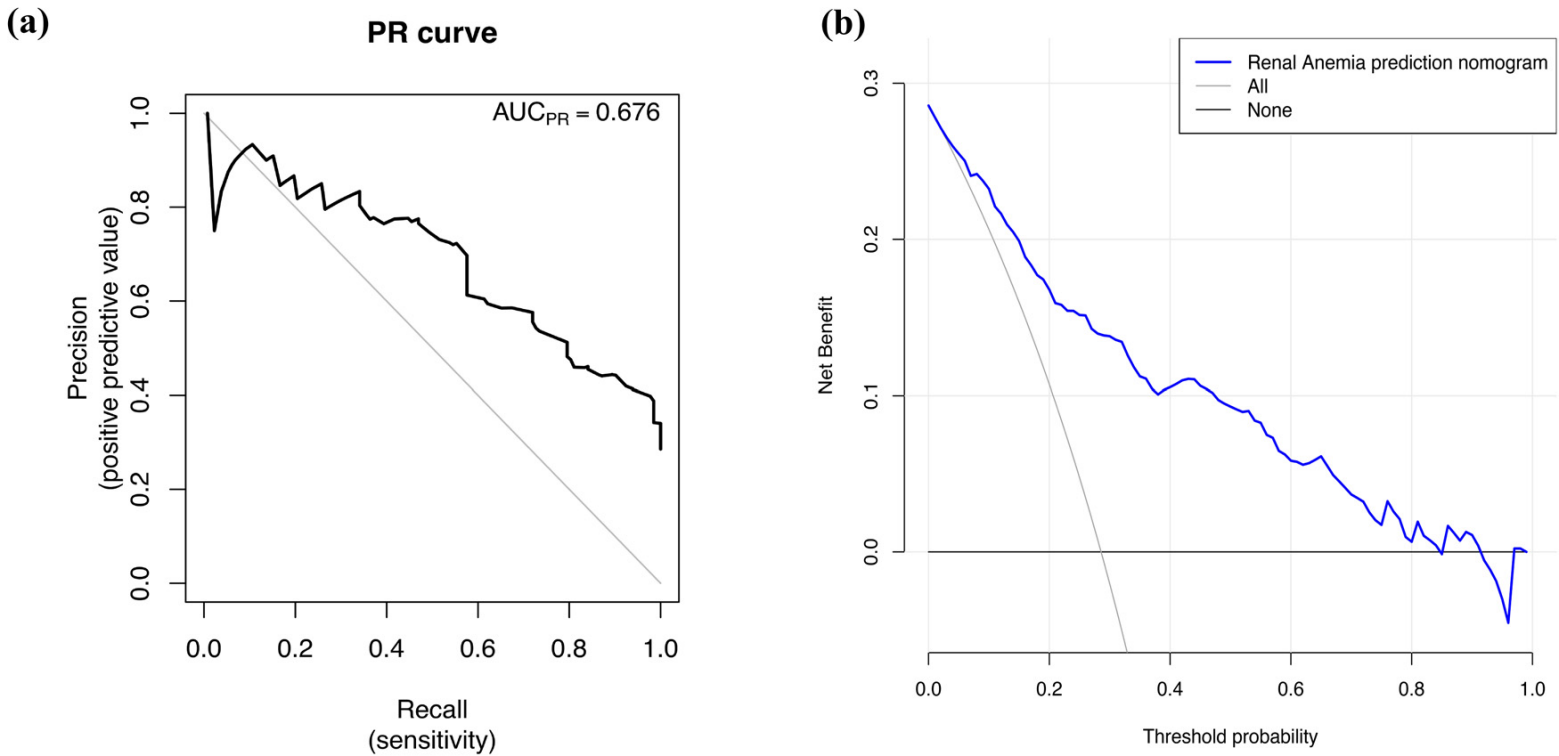
PR curve and decision curve analysis (DCA) for the renal anaemia nomogram. (a) PR curve. It showed that the AUC of this model for predicting renal anaemia was 0.676. (b) DCA. The *y*-axis measures the net benefit. The blue solid line represents the renal anaemia nomogram. The solid grey line represents the assumption that all patients with IgA nephropathy developed renal anaemia. The solid black line represents the assumption that none of the patients with IgA nephropathy developed renal anaemia. The decision analysis curve showed that the net benefit rate was >0 at the high-risk threshold of 1–84%, which was clinically significant. Abbreviations: PR curve, precision-recall curve; AUC, area under the curve.

A model containing the above independent predictors was developed and presented as a nomogram (Figure 2). To apply the nomogram model, the scores of different variables are first obtained on the vertical line on the nomogram. Then, the scores of all variables are added to obtain the total score, which finally allows determination of the corresponding predicted risk value by connecting the prediction line to the total score line at the bottom of the nomogram. The calibration curve for the nomogram used to predict the development of renal anaemia in patients with IgA nephropathy showed good consistency in this cohort (Figure 3a). The C-index of the cohort was 0.848 (95% confidence interval: 0.811–0.885), while the area under the ROC curve was 0.835 (Figure 3b), the sensitivity was 0.72, the specificity was 0.79, the positive predictive value (PPV) was 0.58, the negative predictive value (NPV) was 0.88, and the area under the PR curve was 0.676 (Figure 4a), suggesting that the model has good discrimination and diagnostic efficacy. An internal validation set was evaluated by bootstrap resampling, and the calculated corrected C-index reached 0.823, suggesting that the good predictive accuracy of the model was preserved. For this nomogram, visualization of the prediction model can indicate a better predictive value.

### 3.5 Clinical application

The DCA of the renal anaemia nomogram is shown in Figure 4b. DCA showed that the net benefit rate was >0 at a high-risk threshold between 1 and 84%, which was clinically significant, and that the smaller the threshold was between 1 and 84%, the higher the net benefit rate.

## 4 Discussion

With the change in the medical model from evidence-based medicine to precision medicine, the latter has rapidly become the focus of attention of the global medical community. The era of big data provides unlimited possibilities for the realization of individualized medicine, in which treatment plans can be tailored according to the individual characteristics of each patient. Clinical prediction models are increasingly widely used in clinical diagnosis, treatment decisions, and patient prognosis management through comprehensive statistical analyses of various clinical data and have become increasingly important as the value of clinical risk prediction and benefit assessment has increased [24]. The nomogram is a graph with high- and low-score lines based on multiple clinical indicators; it is based on multivariable regression analysis, can be used to predict a certain clinical outcome or adverse event rate, and is one of the most widely used statistical methods in clinical research [25]. Moreover, it has visual and mathematical advantages and facilitates the probability calculation of risk factors or other predictor variables in clinical practice [26]. This study is the first to apply a nomogram to the risk study of IgA nephropathy complicated by renal anaemia.

We obtained nine variables that are easy to apply clinically and used them to develop and validate a new tool to predict the risk of IgA nephropathy complicated by renal anaemia. Risk factors extracted from among demographic characteristics, laboratory findings, and pathological findings were included in the nomogram for the individualized prediction of disease occurrence. Internal validation of the cohort data showed that the model had good discrimination and calibration ability, especially via its high C-index, indicating that the model can be widely and accurately used in a large number of clinical samples. These nine variables were age, sex, DBP, ALB, CHOL, TG, CKD stage, M, and T, which were associated with IgA nephropathy complicated by renal anaemia. The nomogram suggests that age ≥60 years (score = 43), female sex (score = 31), DBP ≤69 mm Hg (score = 36), ALB ≤30 g/L (score = 43), serum CHOL <5.72 mmol/L (score = 23), serum TGs <1.7 mmol/L (score = 23), CKD stage 3 or higher (score = 30 for CKD stage 3 and score = 100 for CKD stages 4–5), mesangial hypercellularity (M1, score = 7), and tubular atrophy/interstitial fibrosis (score = 5 for T1 and score = 38 for T2) may be key factors in determining renal anaemia in patients with IgA nephropathy.

In this study, we found that age ≥60 years was an independent predictor of renal anaemia in patients with IgA nephropathy, similar to the study by Melissa E. Stauffer, in which the incidence of anaemia tended to increase with increasing age in elderly CKD patients [4]. The reason for this is two-fold. First, with age, the kidney changes morphologically and functionally. Renal ageing is morphologically characterized by the gradual loss of nephrons, glomerulosclerosis, tubular atrophy, renal interstitial fibrosis, and arteriosclerosis, while functional changes mainly consist of reduced renal effective plasma flow and decreased eGFR [27,28]. Second, elderly patients have notably poor prognostic factors, such as decreased eGFR, massive proteinuria, a high number of comorbidities, and relatively severe renal chronicity [29]. Another independent risk factor we identified was female sex. Oh et al. found that in IgA nephropathy, female sex was strongly associated with decreased haemoglobin [30]. Poudel et al. also found a high incidence of anaemia in women with CKD [31]. The specific reasons for the difference in anaemia between sexes are still unclear; one possibility is that hepcidicin, which is increased in postmenopausal women and constant with age in men, aggravates chronic disease-related anaemia at high levels [32]. Therefore, doctors should pay special attention to female patients with anaemia in clinical work, and sex should also be considered in the clinical observation and intervention of patients with renal anaemia. In terms of blood pressure, we found that a lower DBP was associated with anaemia, which is speculated to be related to the frequent occurrence of increased pulse pressure in patients with vascular calcification in CKD. Vascular calcification is also another common complication of CKD [33]. Liu et al. also found that orthostatic hypotension in CKD is closely related to haemoglobin reduction [34]. Lower ALB, serum CHOL, and TGs are key factors in anaemia and may represent a state of malnutrition in the body, which is also closely related to anaemia because of chronic inflammation in CKD [7,35]. Investigation of the interaction between nutritional markers and inflammatory cytokines or adipokines is necessary to understand the development of anaemia in CKD.

In the nomogram model, the other two factors accounting for the higher weight score and that were independent predictors of concurrent renal anaemia in patients with IgA nephropathy were patients with CKD stages 3–5 and pathological findings of more severe tubular atrophy/interstitial fibrosis (T2). We demonstrated that the prevalence of anaemia increases with CKD stage, similar to previous studies [11]. We also found that patients with stage 3 CKD need to be monitored for renal anaemia and possibly undergo intervention. This is in line with the results of the study by Jha et al., who found that patients with stage 3 CKD complicated by anaemia (<13 g/dL in men or <11 g/dL in women) had an equivalent risk of progression to end-stage renal disease to non-anaemic patients with stage 4 CKD, further indicating the need for early intervention in patients with CKD complicated by anaemia [36]. The reason for this may be related to the fact that tubular atrophy/interstitial fibrosis injury can reach more than 50% (T2) in patients with IgA nephropathy, which is the link between patients with stages 3–5 CKD and renal anaemia. In further analysis, an important cause of renal anaemia is the insufficient production of EPO by cells called erythropoietin-producing cells (REPs). Numerous studies have shown that REPs are intrinsically renal interstitial fibroblasts [37,38,39], and hypoxia is a switch for their activation, regulating EPO production through the PHD2-HIF2α-EPO signalling pathway [5]. More interestingly, it has been found that REPs transform into myofibroblasts upon kidney injury, leading to the development of renal interstitial fibrosis [40]. Renal interstitial fibrosis in CKD is closely related to renal anaemia, which inspires us to slow the further progression of CKD by targeting and regulating the cellular characteristics of REPs while preventing renal anaemia.

In addition, we analysed ROC and PR curves to validate the nomogram model and evaluated the clinical usability and benefits of the prediction tool through DCA, which suggested that this model can be applied to larger clinical samples. However, this study has some limitations. First, our study is a single-centre study, and the model may require further external validation through a multicentre sample study. Second, the risk factors did not include all potential factors affecting renal anaemia, and thus the model may be somewhat biased.

In conclusion, we constructed a nomogram model for predicting the risk of renal anaemia in patients with IgA nephropathy. The model achieved a reasonable accuracy, discrimination, and predictive ability, indicating its potential usefulness for the clinical screening of high-risk patients and the development of more targeted intervention strategies.
